# The obesity paradox in multiple myeloma: A report from Multiple Myeloma Research Foundation (MMRF) dataset

**DOI:** 10.1002/cam4.6685

**Published:** 2023-11-06

**Authors:** Liang Ren, Tianhong Xu, Yuhong Ren, Peng Liu

**Affiliations:** ^1^ Department of Hematology Zhongshan Hospital, Fudan University Shanghai P. R. China; ^2^ Cancer Center Zhongshan Hospital, Fudan University Shanghai P. R. China

**Keywords:** BMI, cytogenetic abnormal, multiple myeloma, obesity paradox, prognosis

## Abstract

**Background:**

Obesity is a risk factor for multiple myeloma (MM). However, we still lack knowledge on the clinical course of obese MM patients in a broad view.

**Methods:**

Here, we reviewed 568 MM patients recorded in the Multiple Myeloma Research Foundation (MMRF) coMMpass dataset. Patients were divided into the normal and obese groups according body mass index (BMI) at diagnosis, and then the baseline characteristics, cytogenetic abnormalities, treatment variability, and survival outcomes were evaluated in the obese cohort.

**Results:**

We found no differences in the characteristics when comparing normal and obese MM patients other than more male in the obese part (50.4% vs. 59.9%, *p* = 0.024). Compared with the normal BMI patients, median overall survival (OS) was shorter for obese MM patients but without significant meaning (82.3 vs. 95.3 months, *p* = 0.25). However, in the subgroup analysis, obese MM patients younger than 65 years had significantly inferior OS than that in the normal category (*p* = 0.047). We also found obese MM patients had a higher overall response rate (ORR) compared with normal BMI patients (92.7% vs. 88.6%, *p* = 0.037). Additionally, obese patients seemed to achieve faster best response during first‐line therapy.

**Conclusions:**

Obesity assumes a paradoxical function in the clinical trajectory of myeloma.

## 1 INTRODUCTION

Multiple myeloma (MM) is the second most common hematological malignancy. In the United States, there were 32,270 new MM patients in 2020 as estimated, nearly 12,830 deaths attributed to MM happened.^1^ During the last decade, with novel agents like proteasome inhibitors (PIs), immunomodulatory drugs (IMiDs), monoclonal antibodies, and autologous stem cell transplantation (ASCT) became more accessible, the survival status of MM patients improved a lot. However, at the moment, MM remains incurable.^2^

The etiology of MM still needs to be unveiled. Some potential risk factors for MM had been assessed like radiation exposure and use of hair dyes.^3^, ^4^, ^5^ Obesity was also associated with an increased risk of MM.^6^, ^7^, ^8^ Obesity has become more common in adults. According to WHO, the rates of obesity has pretty much three times that of 1975.^9^ Meanwhile, the incidence of MM also increased over the past decades.^10^ With the excess adipocytes which predominant in adipose tissue accumulated in the bone marrow, this specific bone marrow microenvironment can provide a favorable milieu for myeloma cells.^11^, ^12^ Several studies had reported that obese people had higher incidence of MM than normal people, more than that, higher mortality was also observed in obese MM patients.^13^, ^14^, ^15^ So, the association between obesity and MM trigged many interests.

Obesity is a significant predictor when physicians make decisions in clinical practice. It can influence the efficacy of medications^16^; in another way, the health‐related quality of life can be complicated when added obesity into adverse events. The role of obesity throughout the entirety of the MM process remains poorly understood. Consequently, this study sought to provide a comprehensive examination of the influence of obesity on various aspects of MM, including baseline characteristics, cytogenetic abnormalities, treatment variability, and survival outcomes.

## 2 MATERIALS AND METHODS

### 2.1 Study population

The analytic cohort is based on MMRF CoMMpass Interim Analysis 18. A total of 1143 MM patients were initially included. The inclusion criteria were as follows: (1) MM patients with available height, weight at the time of diagnosis; (2) obesity was identified using body mass index (BMI) calculated by the formula: BMI = weight (kg)/height (m)^2^, and then categorized as: underweight (<18.5), normal weight (18.5–24.9), overweight (25–29.9), obesity (≥30);^17^ and (3) the overall survival (OS) of patients >30 days. These resulted in a study group of 568 MM patients (Figure S1).

The listed parameters were collected: patients' baseline characteristics before treatment, cytogenetic abnormalities, treatment regimens and responses information, and survival outcomes. The final data were reviewed retrospectively.

### 2.2 Statistical analysis

The chi‐square tests or Fisher's tests were conducted to compare categorical variables between different BMI groups. The Kaplan–Meier method was used to estimate progression‐free survival (PFS) and OS, then compared between groups by log‐rank test or two‐stage test. Cox regression analysis was performed to identify risk factors of PFS and OS. All *p*‐values were two‐sided and <0.05 were considered significant. R software (version 4.0.5), SPSS 25.0, and GraphPad Prism 8 were used for statistical analyses.

## 3 RESULTS

### 3.1 Clinical characteristics of the study cohort

The 568 MM patients who met the eligibility criteria for inclusion were studied. The median follow‐up time was 70.5 months (range: 1.5–98.7 months). The median age of the whole group was 64 years (range: 32–93). The baseline characteristics of the selected patients are briefly presented in Table 1. There were more aged patients (≥65) in the normal group (52.8% vs. 46.2%, *p* = 0.119); more male patients were observed in the obese group (59.9% vs. 50.4%, *p* = 0.024). Besides this, none of the clinical factors made a significant difference between the two groups. However, it is noteworthy that nearly all the listed adverse clinical parameters exhibited higher frequencies in the normal weight group than in the obese group, although these differences did not reach statistical significance.

**TABLE 1:** Baseline characteristics of MM patients.

	Normal		Obese		*p*
	*n* = 254	(%)	*n* = 314	(%)	
Age					
≥65	134	52.8	145	46.2	0.119
<65	120	47.2	169	53.8	
Sex					
Male	128	50.4	188	59.9	0.024
Female	126	49.6	126	40.1	
Race					
Black	40	15.7	61	19.4	0.099
White	195	76.8	235	74.8	
Asian	8	3.1	2	0.6	
Other	11	4.3	16	5.1	
ECOG					
0–2	207	81.5	262	83.4	0.137
3–4	17	6.7	10	3.2	
Missing	30	11.8	42	13.4	
Scr (μmol/L)					
Scr > 177	32	12.6	27	8.6	0.11
Scr ≤ 177	217	85.4	285	90.8	
Missing	5	2	2	0.6	
Hb (g/L)					
Hb < 100	109	42.9	117	37.3	0.171
Hb ≥ 100	145	57.1	197	62.7	
LDH (U/L)					
LDH ≥ 250	37	14.6	36	11.5	0.11
LDH < 250	161	63.4	225	71.7	
Missing	56	22	53	16.9	
ALB (g/L)					
ALB < 35	109	42.9	119	37.9	0.108
ALB ≥ 35	138	54.3	192	61.1	
Missing	7	2.8	3	1	
Ca (mmol/L)					
Ca > 2.75	16	6.3	19	6.1	0.153
Ca ≤ 2.75	231	90.9	293	93.3	
Missing	7	2.8	2	0.6	
β2‐MG (mg/L)					
β2‐MG ≥ 5.5	57	22.4	74	23.6	0.187
β2‐MG < 5.5	163	64.2	213	67.8	
Missing	34	13.4	27	8.6	
ISS stage					
Stage I–II	164	64.6	218	69.4	0.245
Stage III	79	31.1	89	28.3	
Missing	11	4.3	7	2.2	

Abbreviations: ALB, albumin; Ca, calcium; ECOG, Eastern Cooperative Oncology Group; Hb, hemoglobin; ISS, International Staging System; LDH, lactate dehydrogenase; Scr, serum creatinine; β2‐MG, serum β2‐microglobulin.

### 3.2 Cytogenetic abnormalities in obese patients

Subsequently, we proceeded to evaluate the status of cytogenetic abnormalities in the cohort of obese patients. As shown in Table 2, the comprehensive distribution of cytogenetic abnormalities did not exhibit any distinct or specific characteristics.

**TABLE 2:** Cytogenetic abnormalities in MM patients.

	Normal	Obese	*p*
	*n* = 254	*n* = 314	
1q21 gain			
Detected	70 (27.6%)	92 (29.3%)	0.76
Not detected	110 (43.3%)	139 (44.3%)	
Unknown	74 (29.1%)	83 (26.4%)	
t(4, 14)			
Detected	22 (8.7%)	26 (8.3%)	0.9
Not detected	166 (65.4%)	211 (67.2%)	
Unknown	66 (26%)	77 (24.5%)	
t(14, 16)			
Detected	9 (3.54%)	10 (3.2%)	0.89
Not detected	179 (70.5%)	227 (72.3%)	
Unknown	66 (26%)	77 (24.5%)	
Del(17p)			
Detected	18 (7.1%)	25 (8%)	0.75
Not detected	162 (63.8%)	206 (65.6%)	
Unknown	74 (29.1%)	83 (26.4%)	
t(6, 14)			
Detected	0 (0)	5 (1.6%)	0.12
Not detected	188 (74%)	232 (73.9%)	
Unknown	66 (26%)	77 (24.5%)	
t(14, 20)			
Detected	2 (0.8%)	5 (1.6%)	0.66
Not detected	186 (73.2%)	232 (73.9%)	
Unknown	66 (26%)	77 (24.5%)	
t(12, 14)			
Detected	2 (0.8%)	3 (1%)	0.9
Not detected	186 (73.2%)	234 (74.5%)	
Unknown	66 (26%)	77 (24.5%)	
t(11, 14)			
Detected	37 (14.6%)	42 (13.4%)	0.81
Not detected	151 (59.4%)	195 (62.1%)	
Unknown	66 (26%)	77 (24.5%)	

### 3.3 The prognostic implications of obesity

**FIGURE 1:**
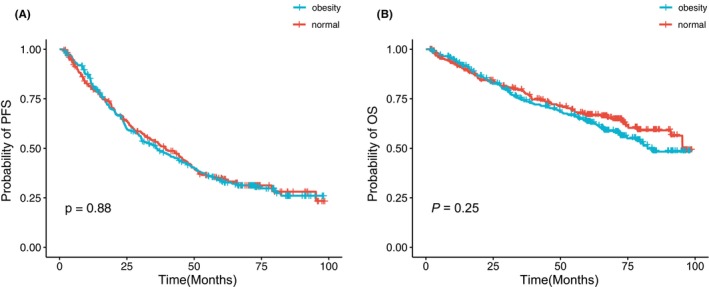
Kaplan–Meier curves for survival in normal and obese MM patients. (A) Progression‐free survival (PFS) and (B) overall survival (OS) in cohorts.

**FIGURE 2:**
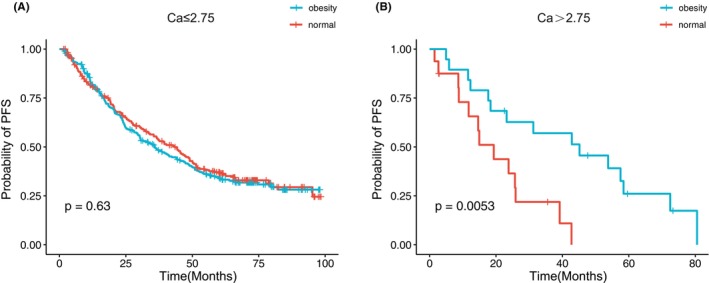
(A, B) Kaplan–Meier curves for progression‐free survival (PFS) in different subgroups. Ca, calcium.

**FIGURE 3:**
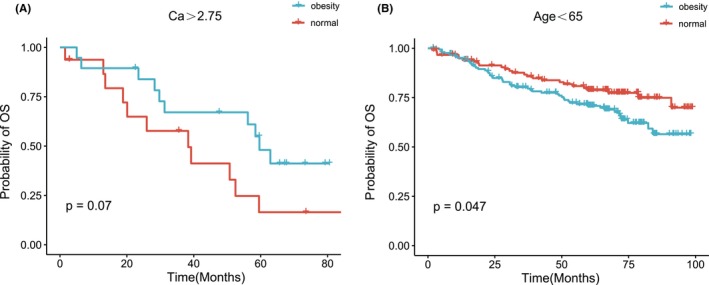
(A, B) Kaplan–Meier curves for overall survival (OS) in different subgroups. Ca, calcium.

The Kaplan–Meier survival analysis revealed no statistically significant differences among the four BMI groups, as illustrated in Figure S2. PFS demonstrated similarity between the normal and obese groups (*p* = 0.88, Figure 1A). Although a trend was observed, indicating shorter survival in the obese category compared to normal MM patients, with median OS of 82.3 and 95.3 months, respectively, the difference did not reach statistical significance (*p* = 0.25, Figure 1B). Survival analyses were conducted based on traditional clinical factors, as shown in Figure S3. Notably, obese MM patients exhibited longer PFS than their normal weight counterparts in the subgroup with Ca > 2.75, not for patients with Ca ≤ 2.75 (median: PFS: 45.1 vs. 19.3 months, *p* = 0.005, Figure 2). OS curves of patients with different variables were plotted in Figure S4. Furthermore, as shown in Figure 3, this extended PFS appeared to translate into prolonged OS, although the statistical significance was not achieved (*p* = 0.07, Figure 3A). Moreover, obesity was able to stratify younger patients (age <65) in terms of survival, with obese individuals experiencing inferior outcomes compared to normal MM patients (*p* = 0.047, Figure 3B).

### 3.4 Therapeutic variability and response

**FIGURE 4:**
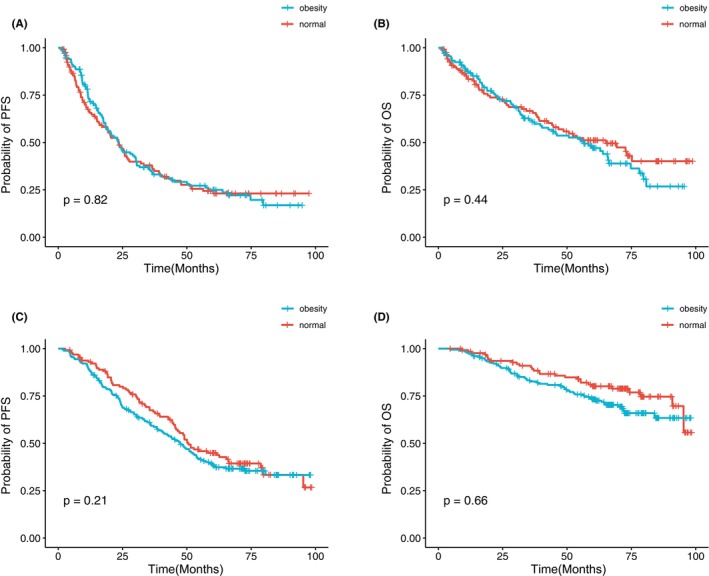
Kaplan–Meier curves for survival stratified by obesity in patients who underwent ASCT or not. (A) Progression‐free survival (PFS) and (B) overall survival (OS) in patients not using ASCT. (C) PFS and (D) OS in patients using ASCT. ASCT, autologous stem cell transplantation.

All patients were treated with at least one novel agent, including PIs or IMiDs, in the frontline therapy (Table 3). In the obese subunit, patients were taking more PIs+IMiDs combination regimens (48.1% vs. 40.9%, *p* = 0.048). The complete response (CR) rate was similar between the two groups (30.7% vs. 29%, *p* = 0.649). When it came to overall response rate (ORR), there were more responders in the obese cohort (92.7%, sCR/CR+VGPR+PR) compared with the normal part (88.6%, sCR/CR+VGPR+PR, *p* = 0.037). MM patients in the obese group seemed to need shorter time to achieve best response no matter scale by cycles or days (*p* < 0.05). More patients were eligible to use stem cell transplant (SCT) in the obese group (69.7% vs. 56.3%, *p* = 0.0009). Out of a total of 254 normal MM patients, 130 underwent ASCT, while among the cohort of 314 obese patients, 179 received ASCT. Furthermore, within the study group, only four individuals underwent allogeneic stem cell transplantation. Upon categorizing the patients based on their ASCT status, our analysis revealed that obesity did not emerge as a discernible risk factor for stratifying MM patients (Figure 4).

**TABLE 3:** Treatment regimens and responses in MM patients.

	Normal	Obese	*p*
	*n* = 254	*n* = 314	
First‐line therapy			
IMiDs only	37 (14.6%)	27 (8.6%)	0.048
PIs only	113 (44.5%)	136 (43.3%)	
PIs+IMiDs combination	104 (40.9%)	151 (48.1%)	
Best‐response after first‐line therapy			
sCR/CR	78 (30.7%)	91 (29%)	0.649
VGPR	102 (40.2%)	140 (44.6%)	
ORR	225 (88.6%)	291 (92.7%)	0.037
SD+PD+unknown	29 (11.4%)	23 (7.3%)	
Cycles to best response			
≤4	65 (25.6%)	86 (27.4%)	0.032
>4	161 (63.4%)	212 (67.5%)	
Unknown	28 (11%)	16 (5.1%)	
Days to best response			
≤252	114 (44.9%)	146 (46.5%)	0.03
>252	112 (44.1%)	152 (48.4%)	
Unknown	28 (11%)	16 (5.1%)	
Stem cell transplant (SCT) eligibility			
Eligible	143 (56.3%)	219 (69.7%)	0.0009
Not eligible	111 (43.7%)	95 (30.3%)	
ASCT	130	179	
Allogeneic	2	2	

Abbreviations: ASCT, autologous stem cell transplantation; CR, complete response; IMiDs, immunomodulatory drugs; ORR, overall response rate; PD, progressive disease; PIs, proteasome inhibitors; PR, partial response; sCR, stringent complete response; SD, stable disease; VGPR, very good partial response.

### 3.5 Cox analysis of related clinical features in MM patients

**FIGURE 5:**
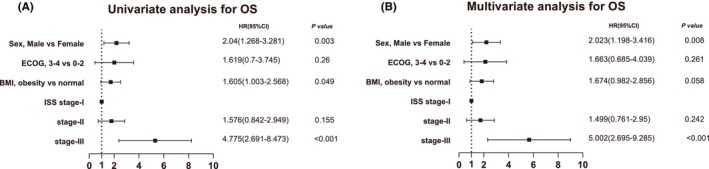
Cox analysis for overall survival (OS) in MM patients younger than 65 years with obesity. (A) Univariate cox analysis for OS (A) and multivariate analysis for (OS) in MM patients who were younger than 65 years. ECOG, Eastern Cooperative Oncology Group; ISS, International Staging System.

Among the entire cohort of patients, certain factors, including age (≥65), male, and ISS stage‐III, exhibited a significant association with inferior PFS and OS in the univariate Cox analysis (*p* < 0.05). Notably, obesity did not demonstrate a substantial impact on OS as a risk factor (*p* = 0.246). These findings were further validated through the multivariate Cox analysis, as indicated in Table S1. Considering the subset of young patients (age <65) who were obese, it was observed that they experienced a shorter OS, as shown in Figure 3B. Consequently, we proceeded to conduct a subgroup analysis focusing on young patients, wherein univariate analysis revealed obesity to be an unfavorable factor (*p* = 0.049, HR: 1.605; 95% CI: 1.003–2.568, Figure 5A). However, obesity was not an independent risk factor in the multivariate Cox analysis (*p* = 0.058, Figure 5B).

## 4 DISCUSSION

In this study, we conducted a comprehensive retrospective analysis to examine the impact of obesity on the clinical features of patients with MM. Our findings revealed no significant differences in clinical features between obese and normal weight groups, except for a higher proportion of male patients in the obese group. Cytogenetic abnormalities did not show a preference for either group.

Regarding outcomes of MM patients, our study demonstrated a trend toward prolonged OS in normal MM patients compared to their obese counterparts, although this trend did not reach statistical significance. Particularly noteworthy is the observation that the adverse impact of obesity on OS was more pronounced in patients younger than 65 years of age. Conversely, obesity appeared to confer a protective effect by PFS in patients with Ca > 2.75. These findings suggest that the influence of obesity on outcomes may vary depending on specific patient characteristics. Intriguingly, obese MM patients demonstrated improved treatment response with higher ORR and quicker responses compared to normal BMI patients.

Previous studies showed that increased mortality was found in obese MM patients. A prospective cohort named Cancer Prevention Study II (CPS‐II) incorporating 900,053 individuals depicted that higher death rate in obese MM patients.^14^ Teras et al^18^ found that young patients (age: 18–21 years) who were obese had higher mortality than normal BMI patients (HR: 1.82; 95% CI: 1.22–2.73). A prospective study tracking mortality among 239,597 participants showed mortality increased along with higher BMI compared to normal BMI patients.^19^ Contradicting these studies, a population‐based study showed that obese MM patients at diagnosis had lower mortality (HR: 0.75; 95% CI: 0.67–0.84) than the normal BMI group, and better OS was observed in the obese‐group (median OS: 35.7 vs. 23.7 months, *p* < 0.001).^20^ Our results found relative inferior OS in obese MM patients although without significant difference. However, among patients who were younger than 65 years, obesity was significantly associated with worse OS. This might indicate young patients have a greater risk of death from MM if they were obese.

We also observed that obese patients had higher ORR. Beyond that, MM patients who were obese needed shorter time to achieve best response or less cycles with first‐line therapy based at least one PIs or IMiDs. Obesity may boost the antitumor efficacy has been reported in many cancers.^21^, ^22^, ^23^ This opposing phenomenon was been termed “obesity paradox.”^24^ In our study, obese MM patients showed better responses to modern therapy. This clinical phenomenon indicated that the present treatment might moderate the adverse effect caused by obesity or even be strengthened in obese patients. However, better responses did not translate into extending OS in our analysis, but rather into a trend toward worse clinical outcomes. This shows the tricky role that obesity plays in the treatment response and long‐term survival of MM patients.

The utilization of ASCT has demonstrated substantial improvements in the survival of MM patients.^25^, ^26^, ^27^ The effect of obesity on the outcomes of MM patients underwent ASCT was controversial. A retrospective study showed obesity BMI at the time of transplantation was an unfavorable factor for OS but not for PFS.^28^ In contrast, a larger cohort found no significant overall effect of obesity on PFS or OS in MM patients undergoing ASCT.^29^ Our study showed that obese MM patients who underwent ASCT made no difference in PFS and OS although the physical conditions of obese patients were more fit for SCT. Obesity did not emerge as a significant variable when considering the use of ASCT versus other treatment modalities, as it did not significantly alter the outcomes of patients undergoing different therapeutic approaches.

Limitations of the retrospective cohort existed in our study. First, certain baseline characteristics of patients were not comprehensively assessed, resulting in an incomplete understanding of the precise status of certain parameters. Furthermore, MM patients included in the MMRF CoMMpass database originated from four different countries, leading to potential variations in laboratory testing protocols and procedures. Consequently, the conclusions drawn from our present study should be interpreted cautiously. A large‐scale investigation, ideally in the form of a prospective research study, is warranted to thoroughly examine the clinical features of MM patients with obesity. Third, the presence of selection bias is a concern as this cohort may have included specific ethnicities, which may limit the generalizability of these results to the broader context of obesity's role in the pathophysiology of MM.

In conclusion, our study found no distinct clinical characteristics associated with obesity in MM patients, except for a higher proportion of male individuals. Importantly, we observed that obesity did not exert an adverse prognostic impact on OS in MM patients, although a trend in that direction was evident. Nonetheless, we noted that obese MM patients under the age of 65 exhibited inferior OS compared to the normal BMI group. Remarkably, the presence of obesity seemed to confer potential benefits in terms of therapy response, as evidenced by higher ORR and more rapid treatment response among obese MM patients. This underscores the paradox role of obesity played in the clinical endings of MM patients. So several aspects concerning the role of obesity in MM remain contentious or unknown. Further investigation is necessary to explore the intricate relationship between obesity and the clinical trajectory of MM.

## AUTHOR CONTRIBUTIONS

**Liang Ren:** Data curation (equal); resources (equal); software (equal); visualization (equal); writing – original draft (equal). **Tianhong Xu:** Formal analysis (equal); methodology (equal); validation (equal). **Yuhong Ren:** Conceptualization (lead); formal analysis (supporting); investigation (equal). **Peng Liu:** Conceptualization (lead); funding acquisition (lead); project administration (equal); supervision (lead); writing – review and editing (lead).

## CONFLICT OF INTEREST STATEMENT

There are no conflicts of interest to declare.

## Supporting information


Appendix S1.

## Data Availability

The dataset analyzed in this study is available in the MMRF CoMMpass Study repository https://research.themmrf.org/.

## References

[1] Siegel RL , Miller KD , Jemal A . Cancer statistics, 2020. CA Cancer J Clin. 2020;70(1):7‐30. 31912902 10.3322/caac.21590

[2] Kumar SK , Dispenzieri A , Lacy MQ , et al. Continued improvement in survival in multiple myeloma: changes in early mortality and outcomes in older patients. Leukemia. 2014;28(5):1122‐1128. 24157580 10.1038/leu.2013.313 PMC4000285

[3] Altekruse SF , Henley SJ , Thun MJ . Deaths from hematopoietic and other cancers in relation to permanent hair dye use in a large prospective study (United States). Cancer Causes Control. 1999;10(6):617‐625. 10616830 10.1023/a:1008926027805

[4] Ichimaru M , Ishimaru T , Mikami M , Matsunaga M . Multiple myeloma among atomic bomb survivors in Hiroshima and Nagasaki, 1950‐76: relationship to radiation dose absorbed by marrow. J Natl Cancer Inst. 1982;69(2):323‐328. 6955540

[5] Preston DL , Kusumi S , Tomonaga M , et al. Cancer incidence in atomic bomb survivors. Part III. Leukemia, lymphoma and multiple myeloma, 1950‐1987. Radiat Res. 1994;137(2 Suppl):S68‐S97. 8127953

[6] Söderberg KC , Kaprio J , Verkasalo PK , et al. Overweight, obesity and risk of haematological malignancies: a cohort study of Swedish and Finnish twins. Eur J Cancer. 2009;45(7):1232‐1238. 19091543 10.1016/j.ejca.2008.11.004

[7] Yang L , Drake BF , Colditz GA . Obesity and other cancers. J Clin Oncol. 2016;34(35):4231‐4237. 27903157 10.1200/JCO.2016.68.4837

[8] Lichtman MA . Obesity and the risk for a hematological malignancy: leukemia, lymphoma, or myeloma. Oncologist. 2010;15(10):1083‐1101. 20930095 10.1634/theoncologist.2010-0206 PMC3227901

[9] Xu K , Tian X , Oh SY , et al. The fibroblast Tiam1‐osteopontin pathway modulates breast cancer invasion and metastasis. Breast Cancer Res. 2016;18(1):14. 26821678 10.1186/s13058-016-0674-8 PMC4730665

[10] Cowan AJ , Allen C , Barac A , et al. Global burden of multiple myeloma: a systematic analysis for the global burden of disease study 2016. JAMA Oncol. 2018;4(9):1221‐1227. 29800065 10.1001/jamaoncol.2018.2128 PMC6143021

[11] Caers J , Deleu S , Belaid Z , et al. Neighboring adipocytes participate in the bone marrow microenvironment of multiple myeloma cells. Leukemia. 2007;21(7):1580‐1584. 17377589 10.1038/sj.leu.2404658

[12] Fairfield H , Dudakovic A , Khatib CM , et al. Myeloma‐modified adipocytes exhibit metabolic dysfunction and a senescence‐associated secretory phenotype. Cancer Res. 2021;81(3):634‐647. 33218968 10.1158/0008-5472.CAN-20-1088 PMC7854508

[13] Wallin A , Larsson SC . Body mass index and risk of multiple myeloma: a meta‐analysis of prospective studies. Eur J Cancer. 2011;47(11):1606‐1615. 21354783 10.1016/j.ejca.2011.01.020

[14] Calle EE , Rodriguez C , Walker‐Thurmond K , Thun MJ . Overweight, obesity, and mortality from cancer in a prospectively studied cohort of U.S. adults. N Engl J Med. 2003;348(17):1625‐1638. 12711737 10.1056/NEJMoa021423

[15] Reeves GK , Pirie K , Beral V , Green J , Spencer E , Bull D . Cancer incidence and mortality in relation to body mass index in the Million Women Study: cohort study. BMJ. 2007;335(7630):1134. 17986716 10.1136/bmj.39367.495995.AE PMC2099519

[16] Blouin RA , Kolpek JH , Mann HJ . Influence of obesity on drug disposition. Clin Pharm. 1987;6(9):706‐714. 3315402

[17] Obesity: preventing and managing the global epidemic. Report of a WHO consultation. World Health Organ Tech Rep Ser. 2000;894:i‐xii. 1–253. 11234459

[18] Teras LR , Kitahara CM , Birmann BM , et al. Body size and multiple myeloma mortality: a pooled analysis of 20 prospective studies. Br J Haematol. 2014;166(5):667‐676. 24861847 10.1111/bjh.12935 PMC4134758

[19] Sonderman JS , Bethea TN , Kitahara CM , et al. Multiple myeloma mortality in relation to obesity among African Americans. J Natl Cancer Inst. 2016;108(10):djw120. 27147231 10.1093/jnci/djw120 PMC5858251

[20] Beason TS , Chang SH , Sanfilippo KM , et al. Influence of body mass index on survival in veterans with multiple myeloma. Oncologist. 2013;18(10):1074‐1079. 24048366 10.1634/theoncologist.2013-0015 PMC3805147

[21] Assumpção JAF , Pasquarelli‐do‐Nascimento G , Duarte MSV , Bonamino MH , Magalhães KG . The ambiguous role of obesity in oncology by promoting cancer but boosting antitumor immunotherapy. J Biomed Sci. 2022;29(1):12. 35164764 10.1186/s12929-022-00796-0 PMC8842976

[22] Gallo M , Adinolfi V , Barucca V , et al. Expected and paradoxical effects of obesity on cancer treatment response. Rev Endocr Metab Disord. 2021;22(4):681‐702. 33025385 10.1007/s11154-020-09597-y

[23] O'Connell F , O'Sullivan J . Help or hindrance: the obesity paradox in cancer treatment response. Cancer Lett. 2021;522:269‐280. 34534616 10.1016/j.canlet.2021.09.021

[24] Gruberg L , Weissman NJ , Waksman R , et al. The impact of obesity on the short‐term and long‐term outcomes after percutaneous coronary intervention: the obesity paradox? J Am Coll Cardiol. 2002;39(4):578‐584. 11849854 10.1016/s0735-1097(01)01802-2

[25] Al Hamed R , Bazarbachi AH , Malard F , Harousseau JL , Mohty M . Current status of autologous stem cell transplantation for multiple myeloma. Blood Cancer J. 2019;9(4):44. 30962422 10.1038/s41408-019-0205-9 PMC6453900

[26] Bergin K , Wellard C , Augustson B , et al. Real‐world utilisation of ASCT in multiple myeloma (MM): a report from the Australian and New Zealand myeloma and related diseases registry (MRDR). Bone Marrow Transplant. 2021;56(10):2533‐2543. 34011965 10.1038/s41409-021-01308-8

[27] Vaxman I , Visram A , Kumar S , et al. Autologous stem cell transplantation for multiple myeloma patients aged ≥ 75 treated with novel agents. Bone Marrow Transplant. 2021;56(5):1144‐1150. 33273658 10.1038/s41409-020-01159-9

[28] Williams A , Baruah D , Patel J , et al. Prevalence and significance of sarcopenia in multiple myeloma patients undergoing autologous hematopoietic cell transplantation. Bone Marrow Transplant. 2021;56(1):225‐231. 32732941 10.1038/s41409-020-01008-9 PMC7855279

[29] Vogl DT , Wang T , Pérez WS , et al. Effect of obesity on outcomes after autologous hematopoietic stem cell transplantation for multiple myeloma. Biol Blood Marrow Transplant. 2011;17(12):1765‐1774. 21624486 10.1016/j.bbmt.2011.05.005 PMC3175301

