# Supplementary material for: The obesity paradox in multiple myeloma: A report from Multiple Myeloma Research Foundation (MMRF) dataset
Source: Cancer Med. 2023 Nov 6;12(23):21400–7. doi: 10.1002/cam4.6685 (PMC10726779; doi:10.1002/cam4.6685)
Supplement: Supplementary file 1 — Appendix S1. [file CAM4-12-21400-s001.docx]

578 patients Normal：259 Obesity：319

172 patients missing height, weight

MMRF-IA18 cohrt 1143 MM patients

971 patients with available BMI

Normal: 18.5-25 Obesity: ≥30

10 patients With≤30 days OS

568 patients Normal：254 Obesity：314

# Fig S1. Flowchart of cohort selection. BMI, Body Mass Index.

1. (B)

1.00

0.75

Probability of PFS

0.50

~~+~~ underweight

~~+~~ normal

1.00

0.75

Probability of OS

0.50

~~+~~ underweight

~~+~~ normal

0.25 0.25

++++++++

++++

+++++

~~+~~ overweight

~~+~~ obesity

++++++++++

++++++++

++++++

+++ +

++

+++++++

++++

+ ++++++++++++++++++

p = 0.73

~~+++ +~~ ++++++++++++++++++++++++++++++++

++++++++++++++++++++++++++++++++++++++++++++++++++++++++ ++

++++++++++++++++++++

++++++++++ +++

~~+~~ overweight

~~+~~ obesity

+ +++++++++++++++++++

+++++++++++++++++++++++

++++++++++++++++++++++++++++++++++++++++++++++++++++++++++++++++++

++++++++++++++++++++++++++++++++++++++++++++++ ++

++++ +++ ++

++++++++++++++++++++++++++++++++++++++++++++++++++ +++

+++++++ +++++++++++++

+++++++ ++++++++++

p = 0.46

0.00

0 25

50

Time(Months)

75 100

0.00

0 25 50 75 100

Time(Months)

# Fig S2. Kaplan-Meier curves for survival stratified by BMI in MM patients. (A) Progression-free survival (PFS) and

1. Overall survival (OS) in cohorts. BMI, Body Mass Index.

# A B C D

~~+~~ obesity

~~+~~ obesity

~~+~~ obesity

##
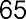

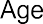
＜

1.00

++

+ +++

+++

++

+

+

+

+ ++ +++

+

+ +++ + ++++++

++++++ ++++++++ ++

++ +++++++++

+ + +++++

+ +++

p = 0.22

~~+~~ normal


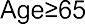


1.00

++++

+++

+++

++

+

+

++++

+

+

++++ ++++

p = 0.42

++++++ +++++

+++++ ++ ++ +

++ ++ +

1.00

+++++

++

+++++

++

++ +

++ +

+ +

+

++++++++ ++++++++ +++

++++++ ++ ++++ ++ ++

p = 0.85

+++

~~+~~ normal


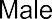


1.00

+

++ +

++

++++

+

+

++++ +

+++

++++++++++++++ ++++ +

p = 1

++++++++ ++ +++ +

~~+~~ normal

0.75

0.75

0.75

0.75

0.50

robability


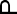
0.25

0.50


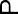
0.25

robability

0.50


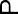
0.25

robability

0.50


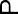
0.25

robability

0.00

0 25 50 75 100

0.00

0 25 50 75 100

0.00

0 25 50 75 100

0.00

0 25 50 75 100

# E F G H

~~+~~ obesity ~~+~~

~~+~~ obesity

~~+~~ obesity

##
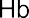

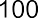

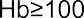
＜

1.00

++

++

+

+

++

+

+

++

+

+ +

+++

+++

+ +++ ++ ++

p = 0.41

+++ ++ +++ ++ + + +

0.75

~~+~~ normal

~~+~~

1.00

++ +

+ ++

+ +++

+

++

+

++

+++ ++

+

+++++++++++ ++++

+++

++ ++ +++ +

++ +

p = 0.78

+

0.75

＜

1.00

++

++++

++++++

+

+

++

+++++ +

+++

++++++++++++ +++++

++++++ ++++ ++++++

p = 0.75

0.75

~~+~~ normal


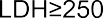


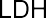

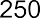
1.00

++

+ + ++

+ +

+

+ + ++++ ++ +

++

+

p = 0.62

0.75

~~+~~ normal

0.50

robability


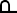
0.25

0.50


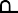
0.25

robability

0.50


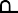
0.25

robability

0.50


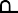
0.25

robability

0.00

0 25 50 75 100

0.00

0 25 50 75 100

0.00

0 25 50 75 100

0.00

0 25 50 75 100

# I J K L


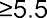


~~+~~ obesity

~~+~~ obesity

~~+~~ obesity

~~+~~ obesity


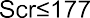


1.00

++

+ ++

+

+++

+++++

+

++

++ +

+++++ +

+ +

+ ++++ +

++++++++ ++++++++++++++ +

++++ +++++

p = 0.84

+++

~~+~~ normal

1.00

++

+

+

+ +++

+ +

p = 0.9

+

++ +

+

+

＞ ~~+~~ normal


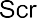

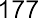

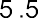
1.00

+++++ +

++

+ +++++++

++

+ +

+++ + ++

+++++++++++++++ ++++

+++++++++ +++++

+

p = 0.66

++

G＜ ~~+~~ normal

1.00

+

+

+

+

+

+

+

++

p = 0.67

+ ++ +++ +

+ + +

+ + + +

+

~~+~~ normal

0.75

0.75

0.75

0.75


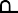
0.50

robability

0.50

robability

0.50

0.50

robability


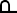
0.25

robability


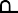
0.25

0.25


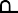
0.25

0.00

0 25 50 75 100

0.00

0 25 50 75 100

0.00

0 25 50 75 100

0.00

0 25 50 75 100

# M N


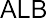

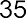
 ~~+~~ obesity

1.00

++

++

+

+++

+++

+ +

+

+ ++++

+

++

+++++ +++++++++++ ++ + ++++

p = 0.74

＜ ~~+~~ normal


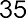


1.00

++ ++

++++

++

+

+

+++ + +

+

+ ++++++ ++++

+++ +++++

p = 0.97

++++ +++ +

+~~++~~

0.75 0.75

0.50 0.50

robability

robability


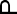

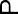
0.25 0.25

0.00

0 25 50 75 100

0.00

0 25 50 75 100

# Fig S3. Kaplan-Meier curves for Progression-free survival (PFS) in different subgroups (A-N). Hb, Hemoglobin; LDH, lactate dehydrogenase; Scr, Serum creatinine; ALB, albumin; β2-MG, serum β2-microglobulin.

(A) (B) (C) (D)

~~+~~ obesity

~~+~~ obesity

~~+~~ obesity

1.00

++++++

++++++

+ +++++++

+++ ++++ +++

++ ++++

+++++++++++

++++++++

+ +++++

+ ++++++ +

++

+++ ++++++++

p = 0.086

0.75

Probability of OS

~~+~~ normal


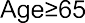


1.00

++++++

+++++ +

+ +

+ + ++ +

++

++ +++

+ +++ +++++++

+++ ++

++++ +++

+++ ++++

+ +

+

p = 1

0.75

Probability of OS

1.00

p = 0.073

0.75

Probability of OS

~~+~~ normal


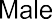


1.00

+++ ++

+++++

++

+ ++

++++++

+++++ +++

+

++++

+++ ++

++++

++++++++++ ++++++++

p = 0.67

0.75

Probability of OS

~~+~~ normal

0.50

0.50

0.50

0.50

0.25

++++ +++++++

++++++++

+ + ++ ++ + +++++++++

++

++++++

+++++++ +

++++ +++++ +

++

++

+++

+ +++++

0.25

0.25

0.25

0.00

0 25 50 75 100

## Time

0.00

0 25 50 75 100

## Time

0.00

0 25 50 75 100

## Time

0.00

0 25 50 75 100

Time

# (E) (F) (G) (H)

~~+~~ obesity ~~+~~

~~+~~ obesity

~~+~~ obesity

##
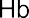

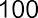

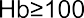
＜

1.00

++++++++

++ +

+++ ++ ++

+

+

+ + +

+++ +++++

+ ++ +

++++

+++ +++++ ++ ++

+ ++

+ + ++

p = 0.095

0.75

Probability of OS

0.50

0.25

~~+~~ normal

~~+~~

1.00

++ ++++

++ ++ +

+++ +++++

+++++++++

++ ++ ++++++++

++++++++++++++ +

+++++ +++ ++

++

p = 0.8

0.75

Probability of OS

0.50

0.25

＜

1.00

+++ +++++++

+ +++++

+++

++++ ++++++++

+++++++++++

+++++ + +++++ ++

+++++++ +++

+ ++++++

p = 0.38

0.75

Probability of OS

0.50

0.25

~~+~~ normal


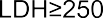


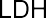

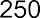
1.00

+++ + +

+

++

+ +

+

+ +

+

+ ++++ +

++ + ++ ++ + +

++

p = 0.47

0.75

Probability of OS

0.50

0.25

~~+~~ normal

0.00

0 25 50 75 100

## Time

0.00

0 25 50 75 100

## Time

0.00

0 25 50 75 100

## Time

0.00

0 25 50 75 100

Time

# (J)

(K) (L)

~~+~~ obesity

~~+~~ obesity

~~+~~ obesity

~~+~~ obesity


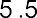

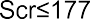


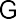


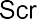

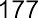
1.00

++++++

++++++++++

++++++++ +

++++ ++++++

+++++++++++++++++++ +

+++++++++++++ ++++

+++++ ++++++

p = 0.34


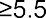


0.75

Probability of OS

~~+~~ normal

1.00

+

+

+

+

+

+ +

+++

++

++

+

+ + +

+ +

p = 0.16

~~+ +~~

0.75

Probability of OS

＞ ~~+~~ normal

1.00

p = 0.25

0.75

Probability of OS

＜ ~~+~~ normal

1.00

p = 0.51

0.75

Probability of OS

~~+~~ normal

0.50

+++ ++++++++

+ +++ +++++

++++++++ +++

++++++++++ ++++ ++++++ +

++++++++++

++++++

+++

+ ++++++++

0.50

0.50

0.50

0.25

0.25

0.25

0.25

0.00

+

+++++

+

++

+

+

+

+++ +

+ ++ ++

+

+ ++ + + ++

+ + +

0 25 50 75 100

## Time

0.00

0 25 50 75 100

## Time

0.00

0 25 50 75 100

## Time

0.00

0 25 50 75 100

Time

# (M) (N)


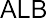

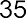
 ~~+~~ obesity ~~+~~

1.00

p = 0.59

＜ ~~+~~ normal


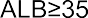


1.00

++++++++

++

++++

++++++

+++++ +++++++

+ +++++++++++++

+++++

+++++ +++

++ ++++++ ++

++++ ++++++

++

p = 0.19

~~+~~ no

0.75 0.75

+++++

+++++

+ +++

+ ++

+ + ++

++

++

+ ++++

+ ++ ++ +++

++++++++++++ +++ ++++

+++++

Probability of OS

Probability of OS

0.50 0.50

0.25 0.25

0.00

0 25 50 75 100

## Time

0.00

0 25 50 75 100

Time

# Fig S4. Kaplan-Meier curves for overall survival (OS) in different subgroups (A-N). Hb, Hemoglobin; LDH, lactate dehydrogenase; Scr, Serum creatinine; ALB, albumin; β2-MG, serum β2-microglobulin.

Univariate analysis Multivariate analysis

PFS OS PFS OS

HR(95%) ***P*** HR(95%) ***P*** HR(95%) ***P*** HR(95%) ***P***

Age

＜65

≥65 1.562(1.269-1.924) ＜0.001 2.083(1.573-2.758) ＜0.001

| 1.649(1.31-2.075) | ＜0.001 | 1.878(1.378-2.56) | ＜0.001 |
| --- | --- | --- | --- |

Sex Female

Male 1.274(1.033-1.572) 0.024 1.778(1.333-2.373) ＜0.001 ECOG

| 1.315(1.04-1.662) | 0.022 | 1.72(1.247-2.373) | 0.001 |
| --- | --- | --- | --- |

0-2

3-4 1.886(1.209-2.940) 0.005 1.311(0.693-2.481) 0.405

| 2.038(1.271-3.266) | 0.003 | 1.422(0.71-2.848) | 0.32 |
| --- | --- | --- | --- |

BMI

normal

obesity 1.017(0.826-1.252) 0.876 1.178(0.893-1.554) 0.246

| 1.136(0.902-1.43) | 0.28 | 1.253(0.923-1.702) | 0.148 |
| --- | --- | --- | --- |

ISS

stage-I

| stage-II | 1.34(1.032-1.74) 0.028 | 1.329(0.909-1.942) | 0.143 | 1.304(0.983-1.73) | 0.066 | 1.311(0.87-1.975) | 0.196 |
| --- | --- | --- | --- | --- | --- | --- | --- |
| stage-III | 1.962(1.509-2.551) ＜0.001 | 3.077(2.171-4.361) | ＜0.001 | 1.858(1.397-2.471) | ＜0.001 | 2.908(1.993-4.244) | ＜0.001 |

Table S1. Progression-free and overall survival in univariate and multivariate Cox analysis in the cohort.
